# A randomised controlled trial of three very brief interventions for physical activity in primary care

**DOI:** 10.1186/s12889-016-3684-7

**Published:** 2016-09-30

**Authors:** Sally Pears, Maaike Bijker, Katie Morton, Joana Vasconcelos, Richard A. Parker, Kate Westgate, Soren Brage, Ed Wilson, A. Toby Prevost, Ann-Louise Kinmonth, Simon Griffin, Stephen Sutton, Wendy Hardeman, on behalf of the VBI Programme Team

**Affiliations:** 1 Behavioural Science Group, Institute of Public Health, University of Cambridge, CB2 0SR Cambridge, UK; 2 PUKCRC Centre for Diet and Activity Research (CEDAR), MRC Epidemiology Unit, University of Cambridge, CB2 0QQ Cambridge, UK; 3 Imperial Clinical Trials Unit, Imperial College London, Stadium House, Wood Lane, W12 7RH London, UK; 4 Health Services Research Unit, Centre for Population Health Sciences, University of Edinburgh, Teviot Place, EH8 9AG Edinburgh, UK; 5 MRC Epidemiology Unit, University of Cambridge, CB2 0QQ Cambridge, UK; 6 Cambridge Centre for Health Services Research, University of Cambridge, CB2 0SR Cambridge, UK; 7 Department of Public Health and Primary Care, University of Cambridge, CB2 0SR Cambridge, UK; 8 School of Health Sciences, University of East Anglia, Norwich Research Park, NR4 7TJ Norwich, UK

**Keywords:** Very brief interventions, Physical activity, Behaviour change techniques, Health promotion, Public health, Primary care

## Abstract

**Background:**

Very brief interventions (VBIs) for physical activity are promising, but there is uncertainty about their potential effectiveness and cost. We assessed potential efficacy, feasibility, acceptability, and cost of three VBIs in primary care, in order to select the most promising intervention for evaluation in a subsequent large-scale RCT.

**Methods:**

Three hundred and ninety four adults aged 40–74 years were randomised to a Motivational (*n* = 83), Pedometer (*n* = 74), or Combined (*n* = 80) intervention, delivered immediately after a preventative health check in primary care, or control (Health Check only; *n* = 157). Potential efficacy was measured as the probability of a positive difference between an intervention arm and the control arm in mean physical activity, measured by accelerometry at 4 weeks.

**Results:**

For the primary outcome the estimated effect sizes (95 % CI) relative to the Control arm for the Motivational, Pedometer and Combined arms were respectively: +20.3 (−45.0, +85.7), +23.5 (−51.3, +98.3), and −3.1 (−69.3, +63.1) counts per minute. There was a73% probability of a positive effect on physical activity for each of the Motivational and Pedometer VBIs relative to control, but only 46 % for the Combined VBI. Only the Pedometer VBI was deliverable within 5 min. All VBIs were acceptable and low cost.

**Conclusions:**

Based on the four criteria, the Pedometer VBI was selected for evaluation in a large-scale trial.

**Trial registration:**

Current Controlled Trials ISRCTN02863077. Retrospectively registered 05/10/2012.

**Electronic supplementary material:**

The online version of this article (doi:10.1186/s12889-016-3684-7) contains supplementary material, which is available to authorized users.

## Background

Physical inactivity is the fourth leading cause of death worldwide and is a key risk factor for non-communicable diseases (NCDs) such as cardiovascular disease, some cancers and type 2 diabetes [1]. The UK government recommendations are for 30 min of at least moderate-intensity physical activity (such as brisk walking or cycling) on at least 5 days per week [2]. However, the majority of adults in the UK do not meet these recommendations [3], and globally physical inactivity is on the rise [1]. In the UK, physical inactivity has been estimated to be directly responsible for 3 % of disability adjusted life years, resulting in an estimated direct cost to the National Health Service of £1.06 billion annually [4]. Worldwide, inactivity is estimated to cause 9 % of premature mortality, comparable to smoking, and if inactivity were decreased by 10 %, more than 533,000 deaths could be averted every year [1].

Given the public health burden associated with sedentary lifestyles, there is a need for scalable, cost-effective interventions to enhance the adoption and maintenance of regular physical activity along the continuum of individual and population-based interventions. One promising avenue is brief and very brief behaviour change interventions in health care settings [5–7]. These have the potential to reach a large proportion of the adult population if delivered in routine primary care consultations or preventive health checks. Review evidence shows that brief interventions in primary care can increase physical activity in the short term when compared with usual care or more intensive interventions [6, 8]. An economic review concluded that brief interventions for physical activity in primary care and the community are cost-effective when longer-term costs and health benefits are considered [9]. However, many so-called brief interventions last up to 30 min [6], which is too long for most primary care consultations [8]. Very brief interventions (VBIs), defined as interventions delivered in a single session of no more than 5 min [6, 10], could be delivered in health checks or similar consultations. However, very few VBIs have been reported in the literature [6, 8, 10], they are poorly described [8, 10], and evidence that they increase physical activity is weak and inconclusive [6]. A systematic review published in 2011 of brief interventions for physical activity in primary care [6] identified only four very brief interventions [11–14], and reported that interventions over 5 min (brief interventions; BIs) increased physical activity more than those under 5 min (very brief interventions). However, no study has directly compared brief with very brief interventions, and overall there is a lack of evidence about whether differential effectiveness is explained by intervention content, mode of delivery or other factors. The descriptions of the BIs and the four VBIs reported in this review [6] were very similar, with both consisting of verbal advice with or without materials (e.g., pamphlets, exercise prescriptions, leisure centre passes) and one or more follow-up components (e.g. visits, phone calls and newsletters); the main difference was the duration. The content of the verbal and written advice given in brief and very brief interventions was also very similar. For example, descriptions of both brief and very brief advice reported by Campbell et al. included such things as: individualized counselling/advice; applying three or more of the 5A’s Behaviour Change Model (ASK, ASSESS, ADVISE, ASSIST, ARRANGE); asking open questions about the benefits of physical activity and barriers to exercise; providing information about the risks of physical inactivity and recommendations for how to increase activity; and providing tailored advice based on stage of motivational readiness to change.

Much of the existing evidence is limited by reliance on imprecise self-report measures of physical activity. Very few studies to date have examined the effect of brief or very brief interventions on objectively measured physical activity [6, 8]. In sum, little is currently known about the effectiveness and cost-effectiveness of VBIs for physical activity.

We developed VBIs to promote physical activity in the context of National Health Service (NHS) Health Checks in England [15], and tested their feasibility prior to the trial reported in this paper [10]. Health Checks target adults between 40 and 74 years and include an assessment of their risk of vascular disease (e.g., type 2 diabetes, heart disease, kidney disease, and stroke) and the offer of appropriate management of risk (e.g. behaviour change support). They are delivered in primary care predominantly by practice nurses and health care assistants, and offer an opportunity to promote physical activity among a large proportion of the adult population.

The VBIs evaluated in the current trial were identified and developed systematically using a two-stage approach. In brief, a short-list of four promising VBIs (a Motivational VBI, an Action Planning VBI, a Pedometer VBI, and a Physical Activity Diary VBI) was identified by the research team, using an iterative approach that combined evidence and expertise from multiple sources (systematic reviews, a scoping review of BCTs, team discussion, stakeholder consultation, a qualitative study, and estimation of resource cost). We then tested the feasibility and acceptability of these promising VBIs among 68 adults attending Health Checks. Further details are reported elsewhere [10]. Using a priori criteria of potential efficacy, acceptability, feasibility and cost, we selected three VBIs: a Motivational VBI, a Pedometer VBI, and a Combined (Motivational and Pedometer) VBI for evaluation in the current trial. A detailed description of the content of each VBI is given in the Methods section.

The aims of this trial were to: 1) assess the potential efficacy, feasibility, acceptability, and cost of these three VBIs against the Health Check alone; and 2) select the most promising VBI for evaluation in a subsequent large-scale randomised controlled trial (referred to in this paper as the ‘main trial’), designed to provide robust estimates of effectiveness and cost-effectiveness.

## Methods

### Trial design

We conducted a randomised controlled trial in which interventions were delivered on a randomised weekly basis and participants received one of three VBIs as part of the usual Health Check consultation: Motivational VBI; Pedometer VBI; Combined VBI; or the Health Check consultation only (Control arm). Physical activity was not measured at baseline as the effects of baseline measurement might have obscured any effects of the VBIs [16]. Furthermore, evidence from pilot work (not reported in this paper) showed that measurement before booking a Health Check appointment reduced Health Check uptake by approximately 50 % and this was not acceptable to the practices and practitioners as Health Checks constitute routine care. Finally, as this is a randomised trial, the groups are expected to be balanced on average for baseline values. Ethical approval was obtained from the UK National Health Service (NHS) National Research Ethics Service (Ref: 12/EE/0200). The trial is registered with Current Controlled Trials (ISRCTN 02863077). The full trial protocol can be accessed at: http://www.phpc.cam.ac.uk/pcu/files/2011/04/VBIWS3-Protocol-V4-0-FINAL.pdf.

### Participants

Participants were recruited between May 2013 and February 2014 from eight NHS primary care practices in urban and rural areas in the East of England. Participants were eligible for the trial if they were eligible for the Health Check: i.e. aged 40–74 years and not previously diagnosed with heart disease, stroke, diabetes, or kidney disease. Participants were excluded if they had no working knowledge of English.

### Procedures

Primary care practitioners (practice nurses and health care assistants) responsible for delivering Health Checks were trained to deliver the VBIs to participants.

Each practice generated a list of 250 eligible patients aged 40–74 years and balanced for gender. Most participants were invited by a letter from the practice to attend the Health Check and take part in the trial, enclosing a Patient Information Sheet. The remainder were recruited by primary care staff who handed out study details to eligible patients in the waiting room. Patients who wanted to take part contacted the practice to book an appointment for a Health Check at a convenient time. Written informed consent for the trial was obtained from each participant by the health practitioner at the start of the Health Check consultation.

An opportunistic sub-sample of participants (two per arm in each practice) was asked for their consent to have their consultation audio-recorded. If the participant gave consent, the Health Check and the VBI (if applicable) were audio-recorded. Following the consultation, participants from this sub-sample who received a VBI were asked to take part in a short face-to-face interview with a researcher. A member of the research team [SP] also conducted face-to-face interviews with each practitioner towards the end of the study.

Four weeks after the Health Check, all participants who attended were sent an accelerometer and a questionnaire which assessed demographic variables, physical activity and beliefs about increasing physical activity. Participants were asked to wear the accelerometer (on the elasticated belt provided) on their right hip for 7 days during all waking hours (except during water-based activities), to complete the questionnaire at the end of the wear-period, and then return both to the research team. Non-responders were telephoned by a researcher after 2 weeks, and recorded as lost to follow up after six failed attempts. Four weeks was selected as the follow-up period as there is evidence that brief interventions may be effective in the short-term, but there is insufficient evidence for their long-term effects [6]; so we would be most likely to observe an effect then.

### Planned interventions

#### The NHS health check consultation

All participants received the usual NHS Health Check, which aims to help prevent heart disease, stroke, type 2 diabetes, kidney disease and certain types of dementia by assessing risk through a combination of personal details, family history of illness, smoking, alcohol consumption, physical activity, BMI, blood pressure and cholesterol. As part of the usual Health Check, all participants should be given support and advice to help them reduce or manage their risk. Participants who were allocated to a VBI condition received the VBI at the end of the usual Health Check procedures. Participants allocated to the Control condition received the usual Health Check only.

#### Practitioner training and promotion of intervention fidelity

Health practitioners who delivered Health Checks underwent a 3-h training session, accompanied by a training manual, to deliver each of the three VBIs. The training session and manual covered: (i) information about study aims and procedures; (ii) information about the importance of promoting physical activity among adults attending Health Checks; (iii) a detailed procedure which described how each component of the VBI should be delivered; (iv) a shortened version of the procedure that practitioners could use as a prompt during the Health Check, to promote fidelity of VBI delivery; (v) a script which gave an example of VBI delivery; (vi) written materials for the participant; and (vii) demonstration and practice of good communication skills to facilitate behaviour change. Two researchers who developed the VBIs introduced the training manual and demonstrated each VBI in role-play. Each practitioner then practised delivering each VBI (role play) and was given feedback on their performance.

#### Intervention delivery

All VBIs involved a very brief face-to-face consultation and written materials for participants (see Additional file 1 for details of the content and component behaviour change techniques (BCTs) of each VBI). The Motivational VBI was composed of 12 BCTs, the Pedometer VBI of seven BCTs, and the Combined VBI of 15 BCTs. All VBIs included goal setting (behaviour), action planning, feedback on behaviour and self-monitoring of behaviour [17]. At the start of each VBI the practitioner gave the participant feedback on their current activity level based on a self-report physical activity assessment as part of the Health Check, and informed the participant about the current UK physical activity recommendations of 30 min of moderate-intensity activity on five or more days of the week.

The three VBIs then followed a different procedure, as outlined below:

#### Motivational VBI

The practitioner: (i) discussed benefits of increasing physical activity; (ii) asked the participant’s view about the importance of increasing physical activity, and their confidence in their ability to do so; and (iii) explained how to use a diary (within the booklet) to set goals, make action plans and self-monitor physical activity. The participant was given a booklet which contained: (i) information on physical activity recommendations; (ii) information about the health, social, environmental and emotional benefits of physical activity; (iii) questions about importance and confidence; (iv) a 4-week physical activity diary encouraging goal setting, action planning, daily self-monitoring of physical activity, goal review, problem solving, and monitoring of emotional consequences; (vi) tips for increasing physical activity and staying motivated (e.g. positive self-talk and mobilising social support); and (vii) information about local physical activity resources.

#### Pedometer VBI

The practitioner explained: (i) the 10,000 steps per day recommendation; (ii) how to wear and use a pedometer to monitor the number of steps walked each day; and (iii) how to use a Step Chart to set a daily step goal (starting with a realistic goal) and record daily steps. The participant was given a pedometer (Yamax SW200 Digi-Walker), a Step Chart and a booklet which contained: (i) information on physical activity and step recommendations; (ii) instructions on how to use the pedometer to monitor daily steps; and (iii) tips for increasing the number of steps.

#### Combined VBI

The practitioner delivered all the components of the Motivational VBI and the Pedometer VBI and gave the participant a Step Chart, pedometer and a booklet that combined information from the Motivational VBI and Pedometer VBI booklets.

### Measures

#### Demographic variables

Age, gender, ethnicity and employment status were collected at baseline (during the Health Check consultation). To reduce the time spent on study procedures during the Health Check, marital status, educational qualifications, occupation group, home ownership and car ownership were assessed through a follow-up questionnaire.

#### Physical activity behaviour

All physical activity outcomes were assessed 4 weeks after the Health Check consultation. The primary outcome was physical activity (total body movement) measured by tri-axial accelerometry (ActiGraph GT3X+, ActiGraph, Pensacola, Florida, USA) expressed as average vector magnitude acceleration (counts per minute). Data collected at 60Hz were integrated into 10-s epochs. Non-wear time, defined as strings of 90 min of consecutive zeros (on the vertical axis) [18], was excluded, and remaining vector magnitude data were summarized into average acceleration (counts per minute (cpm)) and time spent in activity intensity categories. To be included in the analysis, participants needed to contribute at least three valid days of data, defined as wear time of at least 10 h for a valid day.

Secondary outcomes derived from the accelerometer data were: step counts (average step counts per day); and average number of minutes per day spent in sedentary/light activity (<2690 cpm); moderate activity (2690–6166 cpm); vigorous activity (≥6167 cpm); and moderate or vigorous activity (≥2690 cpm) [19].

Self-reported physical activity outcome measures were obtained using the validated Recent Physical Activity Questionnaire (RPAQ) [20]. Total PAEE (Physical Activity Energy Expenditure), domain-specific PAEE (Home, Work, Leisure-time and Commuting), and Screen/TV viewing time over the past 4 weeks were calculated using reported frequency and duration for each activity, together with estimated activity-specific metabolic cost [21].

#### Beliefs about increasing physical activity

The BCTs included in the VBIs (e.g., information about health consequences, social support and graded tasks) were hypothesised to increase physical activity via the following mediators informed by the Theory of Planned Behaviour [22]: intention, attitude, social norms and perceived behavioural control. Therefore, a questionnaire assessed these beliefs with regard to increasing physical activity [22]. *Instrumental Attitude* was measured with two items (Cronbach’s α = 0.49): ‘Being more physically active in the next 4 weeks would be good for me’, and ‘For me, being more physically active in the next 4 weeks would be harmful’. *Affective attitude* was measured with two items (Cronbach’s α = 0.62): ‘For me, being more physically active in the next 4 weeks would be boring’ and ‘For me, being more physically active in the next 4 weeks would be enjoyable’. *Subjective Norm* was measured with two items (Cronbach’s α = 0.49): ‘Most people who are important to me would want me to be more physically active in the next 4 weeks’ and ‘It is expected of me that I will be more physically active in the next 4 weeks’. *Perceived behavioural control* was measured with two items (Cronbach’s α = 0.54): ‘It would be difficult for me to be more physically active in the next 4 weeks even if I wanted to’ and ‘I am confident I could be more physically active in the next 4 weeks, if I wanted to’. *Behavioural intention* was measured with two items (Cronbach’s α = 0.88): ‘It is likely that I will be more physically active in the next 4 weeks’ and ‘I intend to be more physically active in the next 4 weeks’. The items were constructed according to the recommendations by Ajzen [22] and measured on a Likert-type scale ranging from 1 (strongly disagree) to 5 (strongly agree).

#### Feasibility

Feasibility was assessed by (i) calculating VBI duration; (ii) assessing fidelity of VBI delivery (practitioner adherence to each VBI protocol, and contamination); and (iii) interviews with practitioners. A coding framework was developed for the VBI audio-recordings to assess VBI duration, practitioner adherence and contamination. Two researchers (SP, MB) who were blind to physical activity outcomes coded duration and fidelity independently, compared their ratings and resolved any differences. VBI duration was defined as the length of time to deliver the VBI, excluding the rest of the Health Check consultation. VBI fidelity was defined as the presence (coded as 1) or absence (coded as 0) of each VBI component as specified in the VBI protocol (see Additional file 2 for fidelity items). An overall fidelity percentage was calculated for each VBI: the mean percentage of VBI components delivered out of those that should have been delivered.

A member of the research team (SP) who was blind to physical activity outcomes read transcripts of the practitioner interviews and conducted a simple content analysis to determine whether VBIs differed in terms of: (i) ease of delivery and (ii) duration of delivery; and (iii) which VBI practitioners thought should be selected for the main trial.

#### Acceptability

A researcher (SP) who was blind to physical activity outcomes assessed transcripts of semi-structured interviews with participants and practitioners [topic guides are available from the first author] to ascertain: (i) participant views about whether the Health Check was an appropriate time to receive a VBI; (ii) whether VBIs differed in acceptability to participants and practitioners; (iii) their views about how the VBIs could be improved.

#### Cost

A researcher (EW) who was blind to physical activity outcomes calculated the per-participant cost of each VBI from: (i) the cost of participant booklets and equipment (pedometers) estimated from billing records; and (ii) the estimated cost of practitioner time to deliver each VBI, based on the cost of a practice nurse contact at prices from 2013 [23] and the average duration of each VBI.

### Sample size

This trial aimed to provide information on potential efficacy, acceptability and feasibility, to inform selection of one VBI for the main trial. Given that large numbers would be needed to detect a small effect with adequate power in the main trial, and that four arms were tested in this trial, we planned to randomise 64 per intervention arm, and a greater number, 128, to the control group in order to improve precision as this group was involved in all controlled comparisons. This resulted in a total sample size of 320, with 16 control participants and 8 participants per intervention group per practice. With 25 % dropout, there would be 240 followed up (48 per intervention arm and 96 in the control arm). As the SD of the accelerometer counts per minute primary outcome was unknown, we expressed the outcome in terms of standard deviation units (“units”). With sample sizes of 48 and 96, a 95 % confidence interval for a difference in sample means has a width of 0.7 units, based on a standard error of 0.18 units. The calculation was based on a confidence interval approach rather than a power based approach which would be applicable in our subsequent trial. An observed effect of 0.2SD is commonly regarded as a ‘small’ standardised effect size. Very brief interventions may be expected to yield ‘small’ or even smaller effects. With these sample sizes, and the consequent 95 % confidence interval width of 0.7SD, for each VBI, if an observed intervention effect (standardised difference in means) of 0.2SD higher than control were to be observed then this would also provide information that there was 87 % probability of a positive intervention effect size. This is calculated by establishing that 87 % of the normal distribution is positive when the mean is 0.2 and 95 % range, based on observing a 95 % confidence interval (−0.15SD to 0.55SD). If alternatively an observed intervention effect size of 0.1SD were to be observed, this would also provide information that there was 71 % probability of a positive intervention effect, based on observing a 95 % CI centred on 0.1SD with width 0.7SD (−0.25SD to 0.45SD). A positive effect size is defined to be one where the intervention mean exceeds the control mean. The probability of a positive effect size was specified as the summary measure of potential efficacy when informing selection of one VBI for our subsequent trial. The sample size was sufficient to provide a precise estimate of the SD (with the 95 % confidence interval covering +/− 10 % from the estimate) to reliably estimate the necessary sample size for the main trial.

### Randomisation

Within each general practice, each calendar week of Health Checks was randomised to one of the four trial arms, such that all participants scheduled to receive a Health Check consultation in the same week were allocated to the same trial arm. Each practice had a different randomisation order, such that the same intervention did not necessarily take place across all practices in the same week. This was practically easier to implement than individual randomisation. It also helped to reduce contamination across VBIs since practitioners could focus on one intervention each week without having to switch between interventions in short periods. Randomisation of weeks was performed according to an unequal 1:1:1:2 allocation ratio to the Motivational arm, the Pedometer arm, the Combined arm, or the control arm respectively. Invitation letters were sent out in stages rather than all at once. This reduced the likelihood of potential bias arising if more motivated participants were to contact the practice for their Health Check appointment earlier and hence be more likely to be randomised to a particular VBI. Participants were blind to allocation at the time of booking their appointment, as were the practice staff who arranged the appointment. Neither the practitioners nor participants were blind to the arm (control or one of three VBIs) at the time of the appointment. The randomisation list was constructed by programming a simple block randomization routine in R software [24], and was prepared in advance by a trial statistician (RAP) who was independent of trial coordination and intervention delivery.

### Statistical analyses

The data were analysed using IBM SPSS Statistics Version 22. Cronbach’s alpha was used to calculate the internal consistency of each belief measure. Following an intention to treat approach, all participants were analysed in the arm to which they were randomised. Each continuous outcome was analysed using a linear regression model. This provided an estimate and 95 % confidence interval for each pairwise difference between the mean in each intervention group and the mean in the control group. Skewed outcomes were first log-transformed before regression analysis and interpreted as percentage changes relative to the control group. Participants with valid primary outcome data were compared with non-responders to identify if there were any differences in baseline characteristics which could then act as potential confounders. These were then adjusted for as covariates in a secondary sensitivity analysis as an alternative to the assumption of the primary analysis that data are missing completely at random. Adjustment for these covariates instead makes a more plausible assumption that data are missing at random after accounting for these covariates, but is considered a secondary analysis approach because it is driven by the data and is posthoc.

All statistical tests were two-sided and assessed at the 5 % level of significance. The regression results were interpreted using a simple Bayesian framework that is based on 95 % confidence intervals and is appropriate when a decision must be based on a study that has unavoidably low power [25]. The posterior probability of a positive effect for each VBI intervention relative to the control arm was estimated using the 95 % confidence interval for the difference in mean counts per minute between the intervention arm and the control arm. Prior to the trial, an intervention has 50 % potential efficacy, representing equipoise between an intervention and control. Posterior to the trial, a probability above 50 % indicates a greater potential, than not, for an intervention to be efficacious relative to the control arm. Further details about the analysis plan and statistical analyses can be found in the trial protocol: http://www.phpc.cam.ac.uk/pcu/files/2011/04/VBIWS3-Protocol-V4-0-FINAL.pdf.

### Selection of VBI for evaluation in main trial

Seven members of the research team [SS, WH, SP, KM, MB, JM, EW] met to review the findings and select one VBI for evaluation in the main trial. The selection was guided by four criteria (potential effectiveness, feasibility, acceptability and cost) and data collected for each criterion (see Additional file 3 for definitions of all criteria and related measures). At the meeting each team member independently reviewed the evidence to rate each of the three VBIs on a Likert-type scale ranging from 1 to 5 for the four criteria: potential effectiveness (1 = less effective than Health Check only; 5 = more effective than Health Check only), feasibility (1 = not at all feasible; 5 = extremely feasible), acceptability (1 = not at all acceptable; 5 = extremely acceptable) and cost (1 = high cost; 5 = low cost). Criteria ratings were summed to produce a total rating for each VBI, and mean ratings were calculated for each VBI. Given the primary care context, feasibility and acceptability were considered to be of equal importance as effectiveness and cost, and so we chose to give equal weight to the four selection criteria. Each member was asked to write down their best-bet VBI and the team then discussed the evidence and ratings of the VBIs until a consensus was reached.

## Results

### Practitioners and participants

We recruited eight primary care practices (out of 12 practices that were invited to participate) and trained 18 practitioners (nine practice nurses and nine health care assistants) in VBI delivery. All practitioners were female, and none received a personal financial incentive for taking part in the study. Two of the eight practices we recruited were in urban areas and six were in rural areas. The Index of Multiple Deprivation 2015 is the official measure of relative deprivation for small areas (or neighbourhoods) in England based on postcode. A deprivation decile of 1 is the least deprived 10 % of small areas nationally, and 10 is the most deprived 10 % of small areas nationally. Two practices were in decile 3, one was in decile 4, one was in decile 6, two were in decile 8; one was in decile 9; and one was in decile 10. One practice with one trained practice nurse withdrew after recruiting 11 participants (six Combined VBI and five Control).

**Fig. 1:**
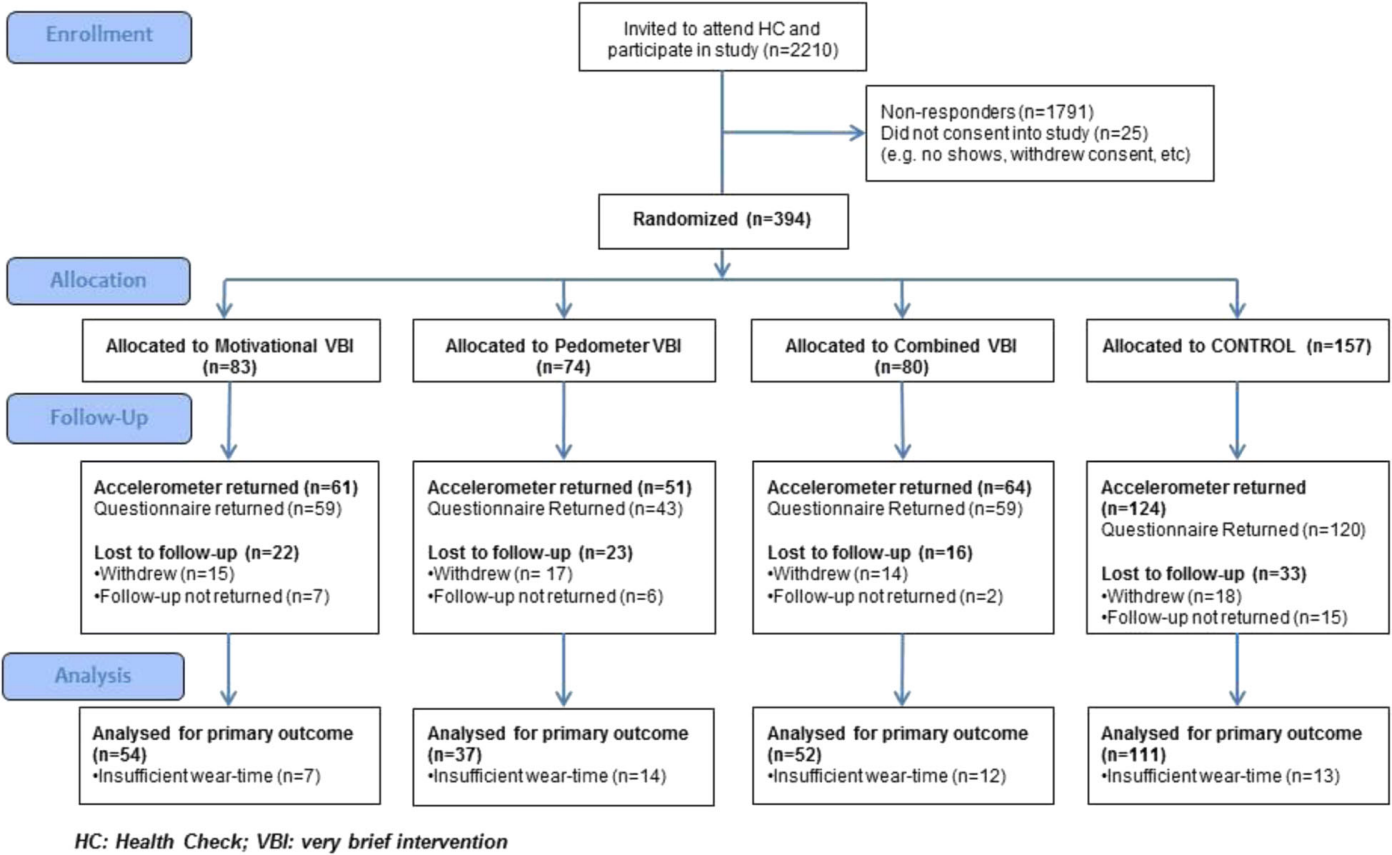
CONSORT diagram

Figure 1 shows the CONSORT diagram. In total, 394 participants were recruited and randomised, and followed up between April 2013 and February 2014. Baseline mean age was 53 years (SD 9.1), 59 % were female, 94 % were white British, and 72 % were employed (see Table 1). At follow-up 84 % were married, 50 % were educated to A-level/Degree, 31 % had a manual occupation, 92 % were home owners and 97 % were car owners. Groups were comparable in terms of age, gender, ethnicity and employment status (Table 1), marital status, qualifications, occupational group, and home and car ownership. One hundred and forty participants (36 %) did not provide a valid primary outcome. The non-response rate varied significantly by arm (*p* = 0.026), being higher in the Pedometer arm (Motivational 29/83 = 35 %; Pedometer 37/74 = 50 %; Combined 28/80 = 35 %; Control 46/157 = 29 %).

**Table 1:** Characteristics of participants (total sample and by trial arm)

Variable	Total Sample	Motivational VBI	Pedometer VBI	Combined VBI	Control (Health Check only)
*N*	394	83	74	80	157
Mean Age (SD), years	52.9 (9.1)	52.1 (8.1)	53.3 (8.4)	51.3 (8.4)	53.9 (10.1)
Gender % female	59 (232)	54 (45)	61 (45)	62 (50)	59 (92)
Ethnicity % white	94 (372)	92 (76)	97 (72)	96 (77)	94 (147)
Employment status % employed	72 (281)	70 (58)	79 (56) ^a^	76 (61)	68 (106) ^a^

Values are % (*n*) unless otherwise specified. ^a^ Missing values for occupation reduced the denominator to 71 in the Pedometer arm and 156 in the Control arm

*VBI* very brief intervention

Fifty-one participants gave consent for audio-recording (Motivational *n* = 11, Pedometer *n* = 13, Combined *n* = 16; Control *n* = 11), and we obtained audio-recordings from all of them. Thirty-seven intervention participants gave consent to be interviewed by a researcher immediately after the Health Check (Motivational *n* = 9, Pedometer *n* = 12, Combined *n* = 16).

### Physical activity behaviour

#### Primary outcome

Sixty-four percent of participants (254/394) provided usable physical activity data. The mean of the primary outcome in this population was estimated from the observed control group mean: 636 (95 % CI: 597–674) counts per minute (Table 2). The standard deviation needed to be able to make sample size calculations for the main trial was 200 cpm, based on the pooled within-group estimate. For the primary outcome the estimated effect sizes (95 % CI) relative to the Control arm for the Motivational, Pedometer and Combined arms were respectively: +20.3 (−45.0, +85.7), +23.5 (−51.3, +98.3), and −3.1 (−69.3, +63.1) counts per minute. Based on these 95 % confidence intervals, we estimated the posterior probability of a positive effect to be 73 % for both the Motivational and the Pedometer VBIs. The probability of a positive effect was estimated to be only 46 % for the Combined VBI, which means that, based on the results from these study participants, there is more likely to be a negative intervention effect than a positive one, indicating a case not to proceed with this intervention. However, for each of the other two interventions, there was increased support for a positive effect (73 % probability) than there was prior to undertaking the study (50 % probability).

**Table 2:** Physical activity at follow-up by arm and comparisons between each VBI and control

	Motivational VBI Mean (95 % CI)	Pedometer VBI Mean (95 % CI)	Combined VBI Mean 95 % (CI)	Control Mean (95 % CI)	Motivational VBI Relative to Control: Comparison of means (95 % CI) ^a^	Pedometer VBI Relative to Control: Comparison of means (95 % CI) ^a^	Combined VBI Relative to Control: Comparison of means (95 % CI) ^a^
Objective physical activity (accelerometer)							
*N*	54	37	52	111			
Counts per minute ^b^	656 (600, 712)	659 (581, 738)	632 (590, 675)	636 (597, 674)	+20.3 (−45.0, +85.7)	+23.5 (−51.3, +98.3)	−3.1 (−69.3, +63.1)
Step counts ^b^	7971 (7252, 8691)	7844 (6921, 8766)	8162 (7464, 8859)	7944 (7370, 8518)	+27 (−894, +949)	−101 (−1155, +954)	+218 (−716, +1151)
Time (min/day) in sedentary/light activity	809.9 (798.6, 821.5)	800.6 (780.2, 821.7)	804.2 (775.7, 833.6)	809.5 (790.8, 828.7)	−1.1 % (−3.9 %, +1.7 %)	−0.7 % (−3.9 %, +2.6 %)	−0.1 % (−2.9 %, +2.9 %)
Time (min/day) in moderate activity	68.4 (61.8, 75.8)	69.7 (59.5, 81.6)	72.1 (65.6, 79.3)	68.7 (62.7, 75.2)	−0.4 % (−13.6 %, +14.8 %)	+1.5 % (−13.7 %, +19.5 %)	+5.0 % (−9.1 %, +21.2 %)
Time (min/day) in vigorous activity	3.4 (2.5, 4.7)	2.5 (1.7, 3.6)	2.6 (2.0, 3.4)	2.5 (2.0, 3.0)	+40.1 % (−1.0 %, +51.6 %)	+1.9 % (−31.5 %, +51.6 %)	+5.3 % (−25.9 %, +49.6 %)
Time (min/day) in moderate or vigorous activity	75.0 (67.7, 83.0)	74.7 (64.2, 86.8)	75.8 (68.6, 83.8)	73.1 (67.0, 79.8)	+2.6 % (−10.7 %, 17.9 %)	+2.2 % (−12.8 %, +19.8 %)	+3.8 % (−9.8 %, +19.5 %)
Self-reported physical activity (Recent Physical Activity Questionnaire, RPAQ)							
*N* ^c^	59	43	59	120			
Total PAEE physical activity energy expenditure(kJ/kg/day)	39.2 (31.5, 48.9)	32.2 (26.7, 38.8)	33.0 (28.3, 38.5)	32.2 (28.2, 36.9)	+21.7 % (−2.9 %, +52.5 %)	−0.2 % (−22.4 %, +28.4 %)	+2.4 % (−18.3 %, +28.3 %)
Home-based PAEE (kJ/kg/day)	2.1 (1.6, 2.8)	2.1 (1.6, 2.8)	2.8 (2.2, 3.5)	2.3 (1.9, 2.8)	−9.5 % (−33.6 %, +23.3 %)	−7.2 % (−34.4 %, +31.1 %)	+19.7 % (−12.1 %, +63.2 %)
Work-based PAEE (kJ/kg/day)	21.2 (16.9, 26.4)	18.4 (14.6, 23.1)	17.9 (14.9, 21.5)	18.2 (15.6, 21.1)	+16.6 % (−9.0 %, +49.4 %)	+1.1 % (−22.7 %, +32.2 %)	−1.3 % (−23.0 %, +26.5 %)
Leisure-based PAEE (kJ/kg/day)	16.5 (12.4, 21.8)	8.7 (5.8, 13.2)	11.0 (8.4, 14.4)	11.0 (8.6, 14.0)	+50.3 % (+2.1 %, +121.2 %)	−20.3 % (−48.3 %, +22.8 %)	+0.7 % (−31.6 %, 48.2 %)
Commuting PAEE (kJ/kg/day)	0.4 (0.2, 0.9)	0.3 (0.1, 0.7)	0.6 (0.3, 1.2)	0.3 (0.2, 0.6)	+29.8 % (−43.2 %, +196.8 %)	−8.5 % (−62.8 %, +125.2 %)	78.4 % (−21.5 %, +305.5 %)
Screen/TV time (hours/day) ^b^	2.71 (2.35, 3.08)	2.72 (2.32, 3.13)	3.11 (2.75, 3.47)	3.04 (2.77, 3.31)	−0.32 (−0.77, +0.12)	−0.31 (−0.81, +0.19)	+0.07 (−0.37, +0.52)

^a^Comparisons are presented unadjusted. Conclusions were unchanged on adjustment for age. ^b^ Values for these variables are means and differences from the control arm mean (with 95 % confidence interval), whereas to account for skewed distributions the PAEE and Time in activity variables are presented as relative percentage increases or decreases compared to the control arm.^c^ Denominators (N) differed for Work based PAEE (79, 44, 35, 44) and Commuting PAEE (77, 44, 34, 45)

PAEE: physical activity energy expenditure

#### Secondary outcomes

The other accelerometer-derived measures of physical activity were similar for all VBI arms relative to control (Table 2).

The self-reported measures (Table 2) were also similar for all VBI arms relative to Control (Table 2), except for reported leisure-based PAEE which was 50.3 % (95%CI: +2.1 %, +121.2 %) higher for Motivational VBI participants (16.5 kJ/kg/day) than Control participants (11.0 kJ/kg/day).

#### Sensitivity analysis

Non-responders were significantly younger than responders (*p* = 0.005) by a mean of 2.7 years, but were no more likely to be male (*p* = 0.67), in paid employment (*p* = 0.16), or of non-white ethnicity (*p* = 0.65). Among responders, there were no significant between-arm differences in gender, ethnicity, or employment. However, responders in the Combined arm were younger than Control arm responders (*p* = 0.032). The sensitivity analysis to adjust between-arm comparisons for age did not appreciably alter confidence interval widths.

### Beliefs about increasing physical activity

All intervention groups reported a stronger intention to be more physically active than control participants (Table 3). The other constructs also generally showed an advantage over the control arm but had low internal consistency.

**Table 3:** Beliefs about increasing physical activity by arm and comparisons between each VBI and control

	Motivational VBI Mean (SD)	Pedometer VBI Mean (SD)	Combined VBI Mean (SD)	Control Mean (SD)	Motivational VBI relative to Control - Difference in means (95 % CI) ^a^	Pedometer VBI relative to Control - Difference in means (95 % CI) ^a^	Combined VBI relative to Control - Difference in means (95 % CI) ^a^
*N*	56	42	57	115			
Instrumental Attitude (Alpha = 0.49) ^b^	4.32 (0.79)	4.23 (0.86)	4.51 (0.55)	4.15 (0.75)	+0.17 (−0.07, 0.41)	+0.07 (−0.19, 0.34)	+0.36 (0.12, 0.59)
Affective Attitude (Alpha = 0.62) ^b^	4.01 (0.82)	3.89 (0.76)	4.10 (0.54) ^c^	3.73 (0.87) ^c^	+0.28 (0.03, 0.53)	+0.16 (−0.12, 0.44)	+0.37 (0.11, 0.62)
Subjective norm (Alpha = 0.49) ^b^	3.31 (1.05) ^d^	3.21 (0.79)	3.26 (0.83)	3.07 (0.83)	+0.24 (−0.05, 0.52)	+0.14 (−0.16, 0.45)	+0.19 (−0.08, 0.47)
Perceived behavioural control (Alpha = 0.54) ^b^	3.71 (0.94)	3.36 (0.94)	3.81 (0.91) ^c^	3.42 (0.90)	+0.29 (−0.01, 0.58)	−0.06 (−0.39, 0.27)	+0.40 (0.10, 0.69)
Behavioural Intention (Alpha = 0.88) ^b^	3.82 (0.90)	3.68 (0.86) ^c^	3.86 (0.82) ^c^	3.46 (0.89) ^c^	+0.36 (0.08, 0.64)	+0.22 (−0.09, 0.54)	+0.40 (0.12, 0.68)

^a^Comparisons are presented unadjusted. Conclusions were unchanged on adjustment for age. ^b^A Cronbach’s alpha coefficient below 0.7 indicates low internal consistency of the two-item scale. ^c^Sample size is one fewer than indicated. ^d^Sample size is two fewer than indicated

### Feasibility

*VBI Duration*: Mean (SD) delivery time was 6 min and 48 s (1 m 51 s) for the Motivational VBI, 5 min and 00 s (1 m 74 s) for the Pedometer VBI, and 9 min and 35 s (2 m 49 s) for the Combined VBI.

*VBI Fidelity*: Mean (SD) overall fidelity ranged from 62 % (18) for the Motivational VBI, 72 % (16) for the Pedometer VBI, and 74 % (10) for the Combined VBI. Contamination was minimal.

*Practitioner views*: Interviews with 12 practitioners (six were unavailable for interview) lasted between 25 and 45 min. Four practitioners reported that ease and duration of VBI delivery depended more on the responsiveness of participants than on VBI content. All practitioners felt that the Pedometer VBI was the easiest and quickest to deliver, and that delivery of the Combined VBI was most difficult and time-consuming (interview themes are available from the first author). Six out of 12 practitioners favoured the Pedometer VBI, five favoured the Combined VBI, and one favoured the Motivational VBI.

### Acceptability

The 12 practitioners interviewed reported that they felt most confident delivering the Pedometer and Combined VBIs and that these two VBIs were the most acceptable to participants and were most likely to be effective. Interviews with 37 participants (lasting 5–15 min) confirmed that the Health Check was a good time to discuss physical activity and that the VBI was a good reminder of the importance of physical activity [interview themes available from the first author]. Participants who received the Motivational or Pedometer VBIs were more likely than participants who received the Combined VBI to rate the physical activity advice given as ‘generic’.

### Cost

The total cost was £6.83 (US$10.06) per participant for the Motivational VBI (£4.99/US$7.35 delivery, £1.84/US$2.71 materials); £17.09 (US$25.17) for the Pedometer VBI (£3.67/US$5.41 delivery, £1.42/US$2.09 materials, £12/US$17.67 pedometer); and £20.98 (US$30.90) for the Combined VBI (£7.03/US$10.35 delivery, £1.95/US$2.87 materials, £12/US$17.67 pedometer).

### Selection of VBI for evaluation in main trial

In order to start the main trial in time to complete it during the grant period, the team had to select the best performing VBI after completion of all participant and practitioner interviews and availability of follow-up data from accelerometers and questionnaires for 62 % of the total sample. The patterns observed for the accelerometer and questionnaire data at the time of the meeting did not differ from those observed in the full dataset. The team reached a consensus that the Pedometer intervention should be evaluated in the main trial as it had the highest combined rating of potential effectiveness, feasibility, acceptability and cost (see Additional file 4) and it was the only VBI deliverable within 5 min. Although the response rate was lower in the Pedometer VBI arm at 1-month follow-up, we found no evidence of a systematic effect of season, practice, age or gender that could explain the differential response rate. Furthermore, we were confident that that the lower response rate was not attributable to some aspect of the Pedometer VBI itself as we would have expected a similarly low retention rate in the Combined VBI arm as the Combined VBI included all the components of the Pedometer VBI.

## Discussion

We aimed to assess the potential efficacy, feasibility, acceptability and cost of three VBIs for physical activity and to select the most promising intervention for evaluation in a subsequent large-scale randomised controlled trial, designed to provide robust estimates of effectiveness and cost-effectiveness. The Motivational and Pedometer VBIs had the greatest potential to increase physical activity compared to the Health Check only (Control), but only the Pedometer VBI could be delivered within 5 min. Participants and practitioners found all VBIs acceptable, and their cost was low. Based on these findings, the Pedometer VBI was selected for further evaluation. Our decision is supported by systematic reviews and meta-analyses of pedometer-based interventions, which showed that they increase physical activity [26, 27].

The Combined VBI had the lowest potential efficacy, perhaps because it contained too many behaviour change techniques (BCTs). This might have reduced its effectiveness [28] by either compromising intervention quality or fidelity of delivery [29], or by overwhelming participants [30].

We observed similar levels of objectively measured or self-reported physical activity when comparing each VBI with the control group. This result is not surprising. First, we did not expect a very brief (5 min) intervention to have medium or large effects on physical activity. Previous trials of very brief interventions for physical activity had inconclusive findings [6], and any effects on physical activity were relatively small. For example, Calfas et al. (1996) [13] found that patients who received 3–5 min of physical activity counselling increased their self-reported walking by approximately five minutes per day. Second, our trial was not powered to detect significant differences between groups but aimed to determine the *potential* efficacy of the VBIs and inform sample size for the main trial. Although the effects of the Motivational and Pedometer VBIs observed in this study are small, the VBIs are scalable, cheap and could be delivered to a large proportion of the adult population, where small effects at the individual level could translate to a substantial public health impact.

Although participants found all VBIs acceptable, practitioners felt most confident delivering the Pedometer and Combined VBIs, and only the Pedometer VBI could be delivered in 5 min. All VBIs commenced with practitioner feedback on current physical activity levels and information about recommendations and how to self-monitor behaviour. However, the Motivational and Combined VBIs also required practitioners to ask participants how they might benefit from increasing their physical activity, how important increasing their physical activity was to them, and how confident they were about increasing their activity, which is likely to have increased the duration of delivery.

### Participant beliefs about increasing physical activity

Our findings suggest that the motivational component of the Motivational and Combined VBIs may have increased participants’ intention to become more active. Although intentions were highest in the Combined VBI group, daily accelerometer counts per minute were lowest in this group. As observed previously [31, 32], increases in intentions do not automatically translate into behaviour change, and inclusion of self-regulation BCTs (such as those included in the Pedometer VBI) may be needed to bridge the intention-behaviour gap [31, 32].

### Limitations

A potential limitation might be that we chose not to measure physical activity (objective or self-report) at baseline. This decision was justified given the evidence for baseline measurement effects and reduced uptake of the Health Checks. Furthermore, our primary purpose in conducting the trial was to compare randomised groups, and statistical inference is still valid in this context, even in the absence of baseline measures. A further potential limitation was the representativeness of our sample. Our participants, being predominantly white and relatively affluent, are representative of the region but not of the UK population as a whole. Finally, non-response rate was unexplainably higher in the Pedometer arm. To improve response rate in the subsequent large-scale trial, we implemented a number of strategies, including sending a text or email reminder of study participation 1 week before sending out accelerometers and follow-up questionnaires [33].

### Strengths

First, our findings are likely to have high ecological and external validity: our study was conducted in a primary care setting; our participants were representative of adults attending NHS Health Checks in the East of England; we recruited practices from rural and urban and from affluent and deprived areas; and we trained health practitioners who routinely delivered NHS Health Checks rather than specialist staff. Second, our randomisation procedure resulted in comparable demographic groups. Third, using a confidence interval approach (Bayesian inference) to determine potential intervention efficacy ahead of the main trial enabled us to evaluate the effect of several interventions relative to control on objectively measured physical activity at the same time, and use the estimates to select the most promising intervention and calculate the sample size for the main trial. Fourth, the use of quantitative and qualitative methods to inform VBI selection, and the use of four a priori defined criteria allowed us to select the best-bet intervention for the main trial based on practicality as well as potential efficacy, and to optimise the intervention ahead of the trial. A key challenge was how to combine the findings for these four criteria. We used a Likert-type rating system in which each research team member rated each VBI on the four criteria and produced an average total rating for each VBI. This method enabled an independent assessment by each research team member, with equal weight given to each criterion.

### Recommendations for research and practice

We would recommend researchers use a confidence interval approach to estimate the potential efficacy of multiple interventions when time and/or resources are limited, as this informs the decision of which intervention(s) warrant further investigation without the need for a much larger study [25]. We demonstrated that all three VBIs are promising in terms of low costs, but it is unlikely that practitioners can deliver the Motivational VBI as part of routine consultations with limited time. Our findings are consistent with systematic review evidence that pedometer-based interventions can increase physical activity [26, 27] and also demonstrate that practitioners can integrate a very brief pedometer intervention into a routine consultation. Hence, it is feasible for practitioners to provide feedback on participants’ current activity levels and encourage goal setting and self-monitoring using pedometers. However, commissioners and policy makers need evidence about cost-effectiveness, therefore large-scale trials are needed to evaluate the effects of VBIs on objectively measured physical activity, supplemented with decision modelling [34] to estimate longer-term costs and outcomes and potential public health impact. We are currently assessing effectiveness and cost-effectiveness of a very brief pedometer intervention based on the Pedometer VBI in a full-scale randomised controlled trial (Current Controlled Trials ISRCTN 72691150).

## Conclusions

This trial showed that three Very Brief Interventions (a Motivational VBI, a Pedometer VBI, and a Combined Motivational and Pedometer VBI) for physical activity in primary care are acceptable and low cost. The Motivational and Pedometer VBIs had the greatest potential efficacy, but only the Pedometer VBI was deliverable within 5 min. The Pedometer VBI was selected for evaluation in a large-scale trial to estimate effects on objectively measured physical activity, cost-effectiveness and potential public health impact.

## Additional files

Additional file 1: Content and component behaviour change techniques of the three very brief interventions. (PDF 219 kb) Additional file 2: Fidelity Coding Items for each very brief intervention used to assess the audio-recorded Health Check consultations. (PDF 192 kb) Additional file 3: Definitions of the four selection criteria, and the measures that informed the ratings of each very brief intervention on each criterion. (PDF 272 kb) Additional file 4: The mean Likert ratings by the research team for each criterion and each very brief intervention. (PDF 428 kb)

## Abbreviations

BCT: Behaviour change technique
BCTTv1: Behaviour change technique taxonomy version 1
BI: Brief intervention
NHS: National Health Service
PAEE: Physical activity energy expenditure
VBI: Very brief intervention

## References

[1] Lee IM, Shiroma EJ, Lobelo F, Puska P, Blair SN, Katzmarzyk PT, Lancet Physical Activity Series Working Group (2012). Effect of physical inactivity on major non-communicable diseases worldwide: an analysis of burden of disease and life expectancy. Lancet.

[2] Department of Health. UK physical activity guidelines. http://www.dh.gov.uk/en/Publicationsandstatistics/Publications/PublicationsPolicyAndGuidance/DH_127931. Accessed 13 Feb 2015.

[3] Chaudhury M, Esliger D, Craig R, Mindell J, Hirani V (2008). Accelerometry in adults. Health survey for England 2008: physical activity and fitness, 1.

[4] Allender S, Foster C, Scarborough P, Rayner M (2007). The burden of physical activity-related ill health in the UK. J Epidemiol Community Health.

[5] National Institute for Health and Care Excellence. Physical activity: brief advice for adults in primary care. NICE public health guidance 44. 2013. https://www.nice.org.uk/guidance/ph44. Accessed 9 Apr 2015.

[6] Campbell F, Blank L, Messina J, Day M, Wood HB, Payne N, et al. Physical activity: Brief advice for adults in primary care. Published Online First. 2012. https://www.nice.org.uk/guidance/ph44/evidence/review-of-effectiveness-and-barriers-and-facilitators-69102685. Accessed 23 Sept 2016.

[7] NICE Public Health Collaborative Centre - Physical Activity. A rapid review of the effectiveness of brief interventions in primary care to promote physical activity in adults. 2006. https://www.nice.org.uk/guidance/ph2. Accessed 23 Sept 2016.

[8] Lamming L, Mason D, Wilson E, Singh GCV, Sutton S, Hardeman W (2012). Very brief interventions to increase physical activity: a systematic review of reviews. Psychol Health.

[9] Vijay GC, Wilson E, Suhrcke M, Hardeman W, Sutton S. Are brief interventions to increase physical activity cost-effective? A systematic review. British Journal of Sports Medicine [In Press] 10.1136/bjsports-2015-094655 PMC4819643 26438429

[10] Pears S, Morton K, Bijker M, Sutton S, Hardeman W (2015). Development and feasibility study of very brief interventions for physical activity in primary care. BMC Public Health.

[11] Bull FC, Jamrozik K (1998). Advice on exercise from a family physician can help sedentary patients to become active. Am J Prev Med.

[12] Lewis BS, Lynch WD (1993). The effect of physician advice on exercise behaviour. Prev Med.

[13] Calfas KJ, Long BJ, Sallis JF, Wooten WJ, Pratt M, Patrick K (1996). A controlled trial of physician counseling to promote the adoption of physical activity. Prev Med.

[14] Marcus BH, Goldstein MG, Jette A, Simkin-Silverman L, Pinto BM, Milan F, Washburn R, Smith K, Rakowski W, Dubé CE (1997). Training physicians to conduct physical activity counseling. Prev Med.

[15] NHS Health Check best practice guidance. 2015. http://www.healthcheck.nhs.uk/commissioners_and_providers/guidance/national_guidance1/. Accessed 23 Sept 2016.

[16] French DP, Sutton S (2010). Reactivity of measurement in health psychology: how much of a problem is it? What can be done about it?. Br J Health Psychol.

[17] Michie S, Richardson M, Johnston M, Abraham C, Francis J, Hardeman W, Eccles MP, Cane J, Wood CE (2013). The behaviour change technique taxonomy (v1) of 93 hierarchically clustered techniques: building an international consensus for the reporting of behaviour change interventions. Ann Behav Med.

[18] Jefferis BJ, Sartini C, Shiroma E, Whincup PH, Wannamethee SG, Lee IM. Duration and breaks in sedentary behaviour: accelerometer data from 1566 community-dwelling older men (British Regional Heart Study). Br J Sports Med. 2014;Sept 17:1–5 10.1136/bjsports-2014-093514 PMC4363289 25232029

[19] Sasaki JE, John D, Freedson PS (2011). Validation and comparison of ActiGraph activity monitors. J Sci Med Sport.

[20] Besson H, Brage S, Jakes RW, Ekelund U, Wareham NJ (2010). Estimating physical activity energy expenditure, sedentary time, and physical activity intensity by self-report in adults. Am J Clin Nutr.

[21] Ainsworth BE, Haskell WL, Whitt MC, Irwin ML, Swartz AM, Strath SJ, O’Brien WL, Bassett DR, Schmitz KH, Emplaincourt PO, Jacobs DR, Leon AS (2000). Compendium of physical activities: an update of activity codes and MET intensities. Med Sci Sports Exerc.

[22] Ajzen I (1991). The theory of planned behaviour. Organ Behav Hum.

[23] Curtis L. Unit Costs of Health and Social Care. Personal Social Services Research Unit, University of Kent at Canterbury, 2013 (p188)

[24] R Core Team (2015). R: A language and environment for statistical computing. R Foundation for Statistical Computing, Vienna, Austria. URL http://www.R-project.org/

[25] Burton PR, Gurrin LC, Campbell MJ (1998). Clinical significance not statistical significance: a simple Bayesian alternative to p values. J Epidemiol Community Health.

[26] Bravata DM, Smith-Spangler C, Sundaram V, Gienger AL, Lin N, Lewis R, Stave CD, Olkin I, Sirard JR (2007). Using pedometers to increase physical activity and improve health: a systematic review. JAMA.

[27] Kang M, Marshall SJ, Barreira TV, Lee J-O (2009). Effect of pedometer-based physical activity interventions: a meta-analysis. Res Q Exerc Sport.

[28] Kinmonth AL, Wareham NJ, Hardeman W, Sutton S, Prevost P, Fanshawe T, Williams K, Ekelund U, Spiegelhalter D, Griffin S (2008). Efficacy of a theory-based behavioural intervention to increase physical activity in an at-risk group in primary care (ProActive UK): a randomised trial. Lancet.

[29] Michie S, Abraham C, Whittington C, McAteer J, Gupta S (2009). Effective techniques in healthy eating and physical activity interventions: a meta-regression. Health Psychol.

[30] Cugelman B, Thelwall M, Dawes P (2011). Online interventions for social marketing health behaviour change campaigns: a meta-analysis of psychological architectures and adherence factors. J Med Internet Res.

[31] Sutton S (1998). Predicting and explaining intentions and behaviour: how well are we doing?. J Appl Soc Psychol.

[32] Hardeman W, Kinmonth AL, Michie S, Sutton S, ProActive Project Team (2009). Impact of a physical activity intervention program on cognitive predictors of behaviour among adults at risk of Type 2 diabetes (ProActive randomised controlled trial). Int J Behav Nutr Phys Act.

[33] Mitchell J, Hardeman W, Pears S, Vasconcelos JC, Prevost AT, Wilson E, Sutton S, VBI Research Team (2016). Effectiveness and cost-effectiveness of a very brief physical activity intervention delivered in NHS Health Checks (VBI Trial): study protocol for a randomised controlled trial. Trials.

[34] Buxton MJ, Drummond MF, Van Hout BA, Prince RL, Sheldon TA, Szucs T, Vray M (1997). Modelling in economic evaluation: an unavoidable fact of life. Health Econ.

