# Supplementary material for: A randomised controlled trial of three very brief interventions for physical activity in primary care
Source: BMC Public Health. 2016 Sep 30;16:1033. doi: 10.1186/s12889-016-3684-7 (PMC5045643; doi:10.1186/s12889-016-3684-7)
Supplement: Additional file 1: — Content and component behaviour change techniques of the three very brief interventions. (PDF 219 kb) [file 12889_2016_3684_MOESM1_ESM.pdf]

Additional file 1: Content and behaviour change techniques of the three very brief interventions

**Content of all very brief interventions (VBIs)**			
**Face-to-face discussion content**	***Practitioner:***<br>• Gives feedback on current physical activity and informs the participant of whether they are meeting the physical activity recommendations.<br>• Asks the participant if they are aware of the Chief Medical Officer's physical activity recommendations ; informs them that the recommendations are for a minimum of 30minutes of moderate-intensity activity on 5 or more days of the week; and emphasises that moderate physical activity is any activity that raises heart rate, breathing or sweating and includes the activities of daily living.		
**VBI-specific content**	**Motivational VBI**	**Pedometer VBI**	**Combined VBI**
**Face-to-face discussion content**	***Practitioner:***<br>• Asks the participant if they can think of any benefits of increasing their physical activity.<br>• Asks the participant questions about the importance of and their confidence for increasing physical activity.<br>• Shows the participant how to use the diary (within the Motivational booklet) to set goals, make action plans and self-monitor physical activity.<br>• Explains that the Motivational Booklet contains tips for how to increase physical activity and stay motivated, and information about local physical activity resources.	***Practitioner:***<br>• Explains the 10,000 steps per day recommendation.<br>• Shows the participant how to use the pedometer to monitor daily steps.<br>• Shows the participant the Step Chart and encourages them to use it to set a daily step goal (starting with a smaller goal) and record daily steps.<br>• Explains that the Pedometer Booklet contains tips for how to increase daily steps.	***Practitioner:***<br>• Explains the 10,000 steps per day recommendation.<br>• Asks the participant if they can think of any benefits of increasing their physical activity.<br>• Asks the participant questions about the importance of and their confidence for increasing physical activity.<br>• Shows the participant how to use diary (within the Motivational booklet) to set goals, make action plans and self-monitor physical activity.<br>• Shows the participant how to use the pedometer to monitor daily steps.<br>• Shows the participant the Step Chart and encourages them to use it to set a daily step goal (starting with a smaller goal at first) and record daily steps.<br>• Explains that the Motivational Booklet contains tips for how to increase physical activity and stay motivated, and information about local physical activity resources.
**Participant materials**	***Motivational VBI Booklet containing:***<br>• Information on physical activity recommendations.<br>• Information about the health, social, environmental and emotional benefits of physical activity.<br>• Questions about the importance of and confidence for increasing physical activity.<br>• A 4- week physical activity diary encouraging goal setting, action planning, self-monitoring, goal review, problem solving, and self-monitoring of emotional consequences.<br>• Tips for increasing physical activity (e.g. positive self-talk, social support).<br>• Information about local physical activity resources.	***Pedometer VBI Booklet containing:***<br>• Information on physical activity recommendations.<br>• Instructions on how to use the pedometer to monitor daily steps.<br>• Tips for how to increase daily steps.<br><br>**Step Chart:** A 4-week chart for setting step goals and monitoring daily steps.<br><br>**Pedometer:** A Yamax Digiwalker SW200.	***Combined VBI Booklet containing:***<br>• Information on physical activity recommendations.<br>• Information about the health, social, environmental and emotional benefits of physical activity.<br>• Questions about importance of and confidence for increasing physical activity.<br>• A 4- week physical activity diary encouraging goal setting, action planning, self-monitoring, goal review, problem solving, and self-monitoring of emotional consequences.<br>• Instructions on how to use the pedometer to monitor daily steps.<br>• Tips for increasing physical activity (e.g. positive self-talk, social support).<br>• Information about local physical activity resources.<br>**Step Chart:** A 4-week chart for setting step goals and monitoring daily steps.<br>**Pedometer:** A Yamax Digiwalker SW200.
**BCTs* included in discussion and materials**	**1.1, 1.2, 1.4, 1.5, 2.2, 2.3, 3.1, 5.1, 5.3, 5.4, 5.6, 15.4**	**1.1, 1.4, 2.2, 2.3, 4.1, 8.7, 12.5**	**1.1, 1.2, 1.4, 1.5, 2.2, 2.3, 3.1, 4.1, 5.1, 5.3, 5.4, 5.6, 8.7, 12.5, 15.4**

*****Numbering refers to BCTTv1 [16]: **1.1** Goal setting (behaviour); **1.2** Problem solving; **1.4** Action Planning; **1.5** Review behaviour goal(s); **2.2** Feedback on behaviour; **2.3** Self-monitoring of behaviour; **3.1** Social support (unspecified); **4.1** Instruction on how to perform the behaviour; **5.1** Information about health consequences; **5.3** Information about social and environmental consequences; **5.4** Monitoring of emotional consequences; **5.6** Information about emotional consequences; **8.7** Graded tasks; **12.5** Adding objects to the environment; **15.4** Self-talk**.**
