# Supplementary material for: A randomised controlled trial of three very brief interventions for physical activity in primary care
Source: BMC Public Health. 2016 Sep 30;16:1033. doi: 10.1186/s12889-016-3684-7 (PMC5045643; doi:10.1186/s12889-016-3684-7)
Supplement: Additional file 2: — Fidelity Coding Items for each very brief intervention used to assess the audio-recorded Health Check consultations. (PDF 192 kb) [file 12889_2016_3684_MOESM2_ESM.pdf]

Additional file 2: Fidelity Coding Items for each very brief intervention used to assess the audio-recorded Health Check consultations

Score 1: Physical Activity Recommendations and Feedback Items (6 Items, assessed for all three VBIs)

1. Practitioner gives the participant feedback on their current physical activity status (e.g. could increase activity/doing plenty already).
2. Practitioner uses 'gentle' language when giving feedback on current physical activity status (i.e. doesn't say 'you're inactive').
3. Practitioner asks participant if they are aware of the physical activity recommendations.
4. Practitioner informs the participant that the recommendations are for 30 minutes of physical activity a day, on most days of the week.
5. Practitioner informs the participant that any activity that elevates heart rate/breathing/sweating counts as physical activity.
6. Practitioner informs the participant that activity is not just 'exercise' and that every-day activities count.

Score 2: Motivational Items (8 Items, assessed for the Motivational VBI and the Combined VBI only)

1. Practitioner asks participant if they can think of any benefits of physical activity.
2. Practitioner works through the Importance questions (in the Motivational booklet) with the participant.
3. Practitioner works through the Confidence questions (in the Motivational booklet) with the participant.
4. Practitioner explains that making a plan and keeping a diary can help the participant increase their physical activity
5. Practitioner shows the participant how to use the diary in the Motivational booklet to set goals, make action plans and self-monitor physical activity.
6. Practitioner explains that there are 4 weeks of blank diary pages in the Motivational booklet for the participant to complete.
7. Practitioner asks participant if they can think of (easy / enjoyable) ways of increasing their physical activity
8. Practitioner encourages the participant to set a goal and/or make a plan.

Score 3: Pedometer Items (8 Items, assessed for the Pedometer VBI and the Combined VBI only)

1. Practitioner explains the 10,000 steps recommendation.
2. Practitioner explains that the pedometer measures steps per day and can be used to measure physical activity.
3. Practitioner shows the participant how to wear and use the pedometer.
4. Practitioner tells the participant that the Pedometer booklet contains instructions for the pedometer, plus information about how to replace the battery.
5. Practitioner explains how to use the step chart included in the Pedometer booklet to monitor daily steps.
6. Practitioner explains how to set a new step goal each week.
7. Practitioner emphasizes that any increase in physical activity is good, not matter how small.
8. Practitioner shows the participant how to convert steps to miles to how to see how far they've walked (on a map of the local area on the back of the step chart).

Score 4: General Items (3 Items, assessed for all three VBIs)

1. Practitioner explains that the rest of the booklet contains other information the participant might find useful (e.g. tips, signposts etc.).
2. Practitioner asks if the participant has any questions.
3. Practitioner encourages the participant to 'give it a try' for the next four weeks.

Overall Fidelity Score

Motivational VBI = Score 1 + Score 2 + Score 4 **(maximum score = 17)**
Pedometer VBI = Score 1 + Score 3 + Score 4 **(maximum score = 17)**
Combined VBI = Score 1 + Score 2 + Score 3 + Score 4 **(maximum score = 25)**
