# Supplementary material for: A randomised controlled trial of three very brief interventions for physical activity in primary care
Source: BMC Public Health. 2016 Sep 30;16:1033. doi: 10.1186/s12889-016-3684-7 (PMC5045643; doi:10.1186/s12889-016-3684-7)
Supplement: Additional file 3: — Definitions of the four selection criteria, and the measures that informed the ratings of each very brief intervention on each criterion. (PDF 272 kb) [file 12889_2016_3684_MOESM3_ESM.pdf]

Additional file 3: Definitions of the four criteria to select very brief interventions and measures used

Criterion	Definition	Measures
1. Effectiveness	Evidence of potential effectiveness to increase physical activity.	• Participant's physical activity obtained from the accelerometer data collected at the 4-week follow-up.
2. Feasibility	The intervention should be deliverable by a health care practitioner within a maximum of 5 minutes as part of an NHS Health Check. It should also be feasible in other respects, e.g. not requiring expensive equipment or extensive specialist training.	• Duration of the very brief interventions (obtained from audio-recordings of the consultation).<br>• Fidelity of the very brief interventions (obtained from audio-recordings of the consultation).<br>• Practitioner semi-structured interviews.
3. Acceptability	The intervention should be acceptable to both practitioners and participants.	• Practitioner semi-structured interviews.<br>• Participant semi-structured interviews.
4. Cost	The intervention should be lowcost.	• Actual cost of printed participant intervention materials and pedometers.<br>• Estimated cost of practitioner time to deliver the very brief interventions: average duration of each very brief intervention obtained from the consultation audio-recordings.
