# Supplementary material for: A randomised controlled trial of three very brief interventions for physical activity in primary care
Source: BMC Public Health. 2016 Sep 30;16:1033. doi: 10.1186/s12889-016-3684-7 (PMC5045643; doi:10.1186/s12889-016-3684-7)
Supplement: Additional file 4: — The mean Likert ratings by the research team for each criterion and each very brief intervention. (PDF 428 kb) [file 12889_2016_3684_MOESM4_ESM.pdf]

Additional file 4: The mean(SD) Likert ratings by the research team (n=7) for each criterion and each very brief intervention

	Effectiveness	Feasibility	Acceptability	Cost	Total
	1 2 3 4 5<br>*Less Effective* ... *More Effective*	1 2 3 4 5<br>*Not at all Acceptable* ... *Extremely Acceptable*	1 2 3 4 5<br>*Not at all Feasible* ... *Extremely Feasible*	1 2 3 4 5<br>*High Cost* ... *Low Cost*	(maximum score =20)
**Motivational VBI**	3.3<br>(1.0)	2.9<br>(0.4)	2.6<br>(0.5)	4.9<br>(0.4)	**13.6**<br>(1.3)
**Pedometer VBI**	3.0<br>(0.0)	4.4<br>(0.5)	4.0<br>(0.6)	3.4<br>(0.8)	**14.9**<br>(0.7)
**Combined VBI**	2.0<br>(0.6)	3.0<br>(1.0)	4.1<br>(0.7)	2.9<br>(1.1)	**11.9**<br>(1.7)
